# *GSTT1*_null_ and rs156697 Polymorphism in *GSTO2* Influence the Risk and Therapeutic Outcome of B-Acute Lymphoblastic Leukemia Patients

**DOI:** 10.3389/fonc.2021.714421

**Published:** 2021-10-14

**Authors:** Shahid M. Baba, Arshad A. Pandith, Zafar A. Shah, Sajad A. Geelani, Javid R. Bhat, Ayaz Gul, Sameer A. Guru, Hamed A. El-Serehy, Abid M. Koul, Sheikh Mansoor

**Affiliations:** ^1^ Department of Immunology and Molecular Medicine, Sher-i-Kashmir Institute of Medical Sciences (SKIMS), Srinagar, India; ^2^ Advanced Centre for Human Genetics, SKIMS, Srinagar, India; ^3^ Department of Clinical Hematology, SKIMS, Srinagar, India; ^4^ Department of Developmental and System Biology, Lurie Children’s Hospital Northwest University, Chicago, IL, United States; ^5^ Department of Zoology, College of Science, King Saud University, Riyadh, Saudi Arabia

**Keywords:** acute lymphoblastic leukemia, glutathione S-transferase, polymorphism, PCR-RFLP, survival analysis, Kashmir, *GSTO1*, *GSTO2*

## Abstract

**Introduction:**

Glutathione S-transferase (GST) gene deletion or polymorphic sequence variations lead to decreased enzyme activity that influences susceptibility and response to chemotherapy in acute lymphoblastic leukemia (ALL). This case–control study investigated the association of GST gene polymorphisms with the etiology and therapeutic outcome of B-ALL among Kashmiri population.

**Methods:**

A total of 300 individuals including 150 newly diagnosed B-ALL patients and an equal number of age and gender matched controls were genotyped for five GST gene polymorphisms by polymerase chain reaction–restriction fragment length polymorphism technique (PCR-RFLP) and multiplex PCR techniques.

**Results:**

Higher frequency of *GSTT1*_null_, *GSTO2-*AG, and *GSTO2-*GG genotypes was observed in ALL cases compared to controls that associated significantly with ALL risk (*GSTT1*_null:_ OR = 2.93, *p* = 0.0001; *GSTO2-*AG: OR = 2.58, *p* = 0.01; *GSTO2*-GG: OR = 3.13, *p* = 0.01). *GSTM1*, *GSTP1*, and *GSTO1* SNPs showed no significant association (*p >* 0.05). Combined genotype analysis revealed significant association of *GSTT1*_null_/*GSTM1*_null_ (OR = 4.11, *p* = 0.011) and *GSTT1*_null_/*GSTP1*-AG (OR = 4.93, *p* = 0.0003) with B-ALL susceptibility. Haplotype analysis of rs4925 and rs156697 revealed that carriers of CG haplotype had increased risk of B-ALL (*p* = 0.04). Kaplan–Meier plots revealed significantly inferior 3-year disease-free survival for *GSTO2*-GG carriers (*p* = 0.002). Multivariate analysis confirmed *GSTO2*-GG as an independent poor prognostic factor for DFS (HR = 4.5, *p* = 0.034). Among combined genotypes, only *GSTT1*_null_/*GSTP1*-AG associated significantly with poorer DFS rates (*p* = 0.032).

**Conclusion:**

This study demonstrated that *GSTT1*_null_ individually or in combination with GSTM1_null_ and *GSTP1*-AG genotypes associated with increased B-ALL risk. Also, rs156697 variant genotypes (AG and GG) associated with B-ALL, whereas the GG genotype of rs156697 influenced the treatment outcome.

## Introduction

Acute lymphoblastic leukemia (ALL) is a complex, multifactorial, and most prevalent hematological malignancy in children comprising almost 25%–30% of all childhood malignancies (1, 2). The etiology of ALL is largely unknown and cannot be ascertained by allelic variability at a single locus. Instead, complex interactions of numerous environmental and genetic components might influence the susceptibility to ALL. During the last decades, multiagent chemotherapy regimens have ensured better prognosis for ALL patients in terms of increased overall survival (OS) rates of approximately 90% and disease-free survival (DFS) close to 80% (3). However, even with the use of more intensive therapy, approximately 20% of the patients do not respond to treatment and succumb either to underlying disease or to toxic side effects of the drugs (4, 5). Genetic and pharmacogenomic studies have suggested that genetic polymorphisms in genes encoding xenobiotic metabolizing enzymes involved in detoxification of carcinogens and drugs modify an individual susceptibility to ALL as well as response to treatment (6–8).

Glutathione S-transferases (GSTs) encoded by 16 genes are members of Phase II detoxification enzyme family that play a pivotal role in cellular detoxification through conjugation of glutathione (GSH) to a large variety of exogenous and endogenous compounds such as chemotherapeutic drugs, carcinogens, and environmental pollutants. Through this process, GSTs facilitate elimination of xenobiotics and protect tissues from being attacked by the reactive electrophiles (9, 10). The genes encoding the glutathione S-transferases comprise eight classes, and in humans, mostly deletion polymorphism of *GSTT1*/*GSTM1* and polymorphic sequence variants of *GSTP1* (rs1695: A>G) and *GSTO1* (rs4925:C>A) and *GSTO2* (rs156697:A>G) transferases have been reported (11–15). These GST functional variants can lead to reduced intracellular enzyme activity resulting in the compromised detoxification of potential carcinogens and increased risk of developing cancer (16). It has also been demonstrated that the human GST enzymes are crucial for the inactivation of anticancer agents like glucocorticosteroids, doxorubicin, adriamycin, vincristine, cyclophosphamide, methotrexate, and 6-mercaptopurine that are used for treating B-ALL (6, 17). Metabolism of anti-cancer drug-like doxorubicin may generate reactive intermediates that can reduce molecular oxygen directly to generate ROS. These species may then react with DNA and macromolecules and trigger toxic responses (18). Glutathione metabolism plays an important role in the detoxification of these electrophilic species and protects cells from oxidative stress (19). GSTs have been shown to influence the resistance to chemotherapy agents either through process of direct detoxification or by inhibiting the MAP kinase pathway (20). Thus, differences in the activity of GSTs may influence the susceptibility as well as prognosis in ALL patients (6, 9, 15, 21). Therefore, an investigation to understand the genetic polymorphic variability at numerous GST gene loci may make it easy to understand the etiology and prognosis of ALL and the detection of individuals at increased risk for developing them.

In literature, number of epidemiological studies have evaluated the role of GST gene polymorphisms in ALL pathogenesis in different ethnic populations, but the results have been conflicting (6, 15, 22). Even few GWAS on ALL have been done in a few populations but their relevance is limited to our population, which is highly ethnic and inbred with conserved gene pool. Furthermore, no GWAS has been reported from our subcontinent in general and North Indian population in particular and whatever has been reported globally have no mention of GST as a susceptibility locus for ALL development. Keeping in view the plausible role of GST gene sequence variations, this investigation was conducted to determine the relation of five different GST gene polymorphic sequence variants with the risk for the development and therapeutic outcome of B-Acute Lymphocytic Leukemia (B-ALL) patients of Kashmir (North India).

## Materials and Methods

### Patients

**Figure 1:**
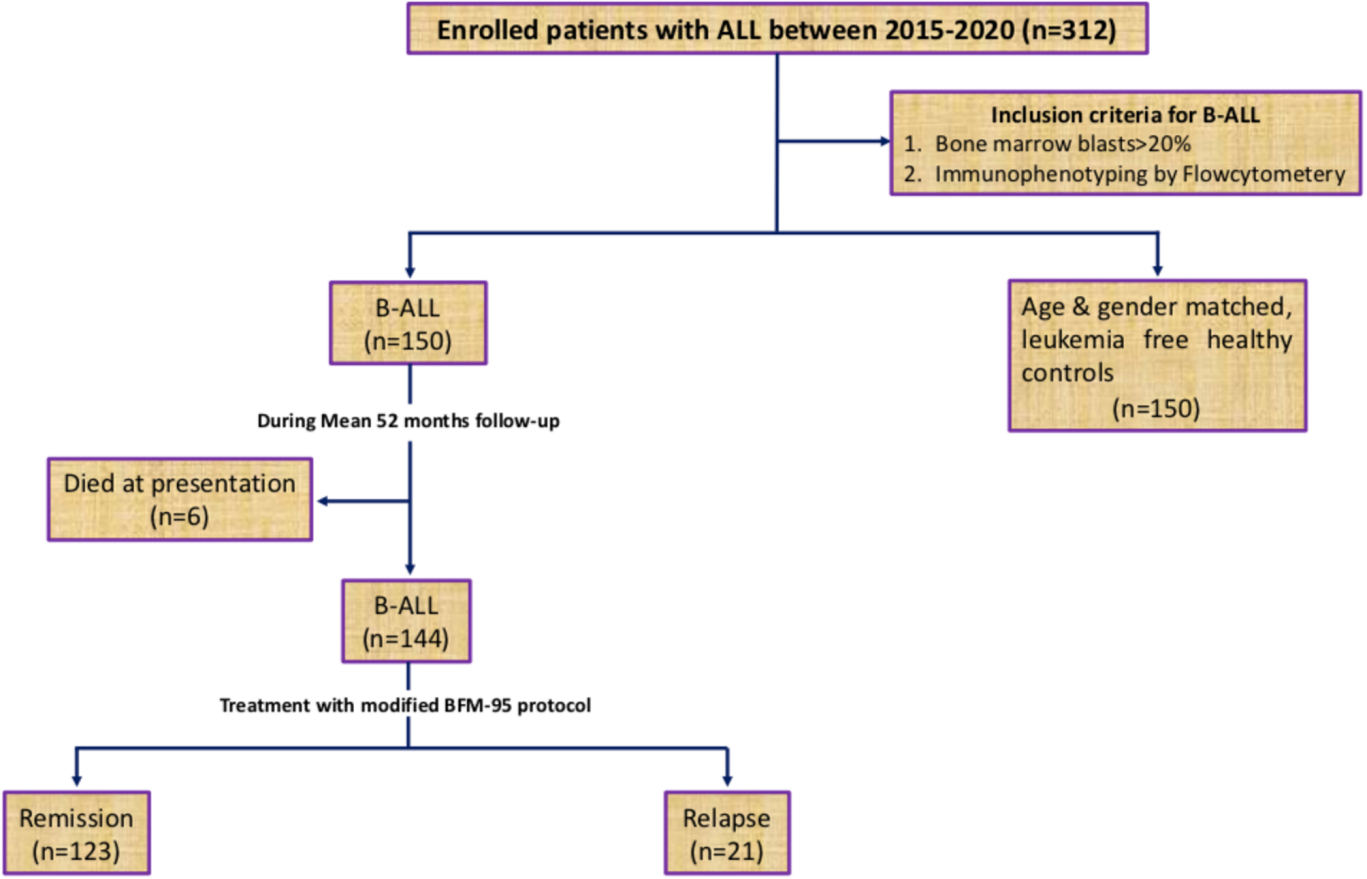
Study design.

The present study conducted in the Department of Immunology & Molecular Medicine, Sher-i-Kashmir Institute of Medical Sciences (SKIMS), J&K (North India) included 150 newly diagnosed B-ALL patients who visited the outpatient clinic or inpatient ward of Department of Clinical Hematology, SKIMS, between May 2015 and September 2019. The inclusion criteria for patient enrolment is shown in **Figure 1**. All patients were assessed clinically and diagnosis was confirmed by determining B-cell subtypes of ALL by immunophenotyping. Peripheral blood/bone marrow cytogenetics was carried out by GTG banding technique according to the International system of human cytogenetic nomenclature 2009 (ISCN, 2009) (23). Details were collected by personal interaction with the patients, and laboratory parameters were recorded from the CBC/bone marrow reports. Patients were risk stratified into low-, medium, and high-risk groups and treated according to modified BFM-95 protocol (24, 25). All the patients were evaluated at baseline, after consolidation, and during as well as after maintenance stage of treatment for their response to chemotherapy. The evaluation of response was based on bone marrow/peripheral blood blast cell counts. Generally, bone marrow leukemic blasts <5% with restoration of normal hematopoiesis was predictive of the attainment of complete molecular remission. Re-emergence of leukemic blasts in the marrow during or after treatment was indicative of leukemic relapse (26). The control group comprised of age and gender matched 150 leukemia free healthy control subjects living in the same geographical area as the patients. This study was conducted only after taking informed consent from all participants or their legal guardians (for patients < 18 years old) and approval from the local Institutional Ethics committee of SKIMS (IEC-SKIMS; Protocol No: 95/2013). All procedures were performed in compliance with the1964 Declaration of Helsinki principles.

### DNA Extraction

Genomic DNA from the peripheral blood/bone marrow leukocytes of patients and peripheral blood of control subjects was extracted using QIAamp DNA Blood Mini Kit (Cat No. 51104), Qiagen (Germany), and its concentration and purity were analyzed by Bio spectrophotometer (Eppendorf AG; Serial No: 6137EQ102539; Germany).

### Multiplex-PCR Analysis for Detection of *GSTM1* and *GSTT1* Genotypes

Multiplex polymerase chain reaction was used for the detection of *GSTM1* and *GSTT1* genotypes using *β-Actin* as internal control. DNA amplification was performed in a thermocycler (Agilent SureCycler 8800, USA) by heating 25 μl of reaction volume containing 50 ng of genomic DNA template, 10× PCR buffer with 20 mM MgCl_2_ (Invitrogen), 50 μM dNTPs (Biotools), 0.5 μM of each primer (Eurofens), and 1.25 U DNA polymerase (Invitrogen) at 94°C for 5 min, followed by 35 cycles of 94°C for 35 s, 54°C for 35 s, and 72°C for 35 s and final extension at 72°C for 7 min. Ten microliters of PCR products was resolved on 2% agarose gel to determine 240-bp, 450-bp, and 868-bp products, respectively, for *GSTM1, GSTT1*, and *β-Actin* alleles (**Supplementary Figure 1A**). Absence of the amplifiable *GSTM1* or *GSTT1* (in the presence of *β-Actin* PCR product) indicated the respective null genotype for each. As PCR cannot differentiate heterozygous and homozygous null genotypes, therefore only double null (−/−) were the null genotypes reported.

### Genotyping of *GSTP1*(rs1695:A>G), *GSTO1*(rs4925: C>A), and *GSTO2*(rs156697: A>G) Gene Polymorphisms

Genotyping of *GSTP1* (rs1695: A>G), *GSTO1* (rs4925: C>A), and *GSTO2* (rs156697: A>G) SNPs was analyzed by polymerase chain reaction restriction fragment length polymorphism (PCR-RFLP). PCR was carried out in 25-μl reaction volume containing similar constituents (except for primers) used for the detection of *GSTT1* and *GSTM1* genotypes. Primer sets and thermal conditions used to amplify the respective PCR products are given in the **Supplementary Table**.

RFLP analysis was done using restriction endonuclease *Alw26I* for *GSTP1* (rs1695: A>G), *Cac8 I* for *GSTO1* (rs4925: C>A), and *MboI* for *GSTO2* (rs156697: A>G) (Fermentas, Germany) according to the manufacturer’s instructions. The digested products were resolved by electrophoresis on 2% agarose gel and visualized on gel doc (Flourchem HD2, USA).

For *GSTP1* (rs1695: A>G) SNP, the wild genotype (AA) lack the restriction site for endonuclease and thereby display an uncut 176-bp band. Two digested bands of 91 bp and 85 bp correspond to the homozygous variant genotye (GG), whereas heterozygous genotype (AG) was represented by three distinct bands of 176 bp, 91 bp, and 85 bp (**Supplementary Figure 1B**).

For *GSTO1* (rs4925: C>A) and *GSTO2* (rs156697: A>G) SNPs, RFLP analysis was performed as described by Pongstaporn et al. (15). The C to A transversion at codon 140 in exon 4 of the *GSTO1* gene was detected after PCR amplification of a 254bp product using specific primer pairs. PCR product was digested with 10 U of *Cac8I* at 37°C for 18 h to produce three different patterns. The wild genotype (CC) demonstrated 186 and 68bp fragments, polymorphic homozygote (AA) presented the uncut 254bp fragment, while the heterozygote genotype (CA) exhibited 254, 186, and 68bp fragments, respectively (**Supplementary Figure 1C**). Similarly, the A to G polymorphism at codon 142 in exon 4 of the *GSTO2* gene was demonstrated by amplification of 185bp PCR product and its digestion with *Mbo*I at 37°C for 18 h to produce three different patterns. The wild-type homozygote (AA) demonstrated an intact 185bp fragment, polymorphic homozygote (GG) exhibited two fragments of 122 and 63bp, while the heterozygote (GA) displayed 185, 122, and 63bp fragments, respectively (**Supplementary Figure 1D**). Genotypes of at least 25% of samples were double blindly reassessed to confirm the results.

### Statistical Analysis

Statistical significance between observed genotype frequencies was calculated according to the Hardy-Weinberg law using SPSS statistics for Windows, Version 25.0, released 2017 (IBM Corp, New York, USA). Data were normalized by Kolmogorov–Smirnov test and was found to be following normal distribution. Numerical data was recorded as median, whereas frequency and percentage were used to express qualitative data. Difference in genotypic frequency distribution between cases and controls was evaluated by Chi-square test. Odds ratio (OR) was calculated as an estimate of relative risk at 95% confidence interval (CI). All the tests applied were two-sided. Cox proportional multivariate hazard model was employed to identify the potential risk factors of all events. OS and DFS analysis of patients according to genotypes was estimated by the Kaplan–Meier method. For all analysis, *p*-values <0.05 was taken as statistically significant.

## Results

The present study successfully genotyped 150 B-ALL patients comprising 92 (61%) males and 58 (39%) females with 126 (84%) cases from the rural and 24 (16%) from the urban dwelling having a median age of 16 years (range 2–58 years) along with 150 age [median 17 years (range 5–62 years)] and gender matched, leukemia free, healthy control subjects for *GSTT1, GSTM1, GSTP1, GSTO1*, and *GSTO2* SNPs. Clinical and laboratory parameters of patients and controls are given in **Table 1**. All polymorphisms were in agreement with the Hardy–Weinberg equilibrium.

**Table 1:** Clinical and laboratory parameters along with risk group categorization of ALL patients and healthy controls.

Particulars	ALL Cases *n* = 150 (%)	Controls *n* = 150 (%)	*p*-value
**Gender**		
Male	92 (61)	86 (57.3)	0.5
Female	58 (39)	64 (42.7)
**Age**		
<20	111 (74)	102 (68)	0.3
≥20	39 (26)	48 (32)
**Dwelling**		
Rural	126 (84)	117 (78)	0.2
Urban	24 (16)	33 (22)
*WBC Count (×10^3^/μl)	15.9 (0.94–248.7)	—	
**Risk Group**			
Low Risk	53 (35)		
Standard Risk	69 (46)	—	
High Risk	28 (19)		
**Outcome**			
Remission	123 (82)		
Relapse	21 (14)	—	
Dead	06 (4)		

*WBC Count (×10^3^/μl) = mean WBC count in ALL patients.

### GST Genotypes Distribution and Risk of B-ALL

A significantly higher frequency of *GSTT1*_null_ genotype 40% was observed in ALL cases compared to 16.0% in healthy controls that associated with increased B-ALL risk (OR = 2.93, 95% CI = 1.55–5.91, *p* = 0.0001) whereas the frequency of *GSTM1*_null_ genotype was 29% in cases compared to 37.3% in controls and showed no significant difference in distribution between the two groups (*p >* 0.05). Also, not only the frequency of *GSTP1* (rs1695: A>G) and *GSTO1* (rs4925: C>A) genotypes but also their respective alleles did not differ significantly between cases and controls (**Table 2**).

**Table 2:** Genotypic and allelic distribution of different GST polymorphisms in ALL patients and controls and their association with B-ALL risk.

Genotypes/Alleles	ALL Cases *n* = 150 (%)	Controls *n* = 150 (%)	OR (95% CI)	*p*-value
***GSTT1***			
Present	90 (60%)	126 (84.0%)	1 (Ref)	
Null	60 (40%)	24 (16%)	2.93 (1.55–5.91)	0.0001^**^
***GSTM1***			
Present	107 (71%)	94 (62.7%)	1 (Ref)	
Null	43 (29%)	56 (37.3%)	0.83 (0.22–1.13)	0.36
***GSTP1* (rs1695:A>G)**				
AA	88 (58.7%)	92 (61.3%)	1 (Ref)	
AG	47 (31.3%)	51 (34.0%)	0.88 (0.07–1.47)	0.66
GG	15 (10%)	7 (4.7%)	1.98 (0.42–5.15)	0.38
Frequency of A	223 (74.3%)	235 (78.3%)	1 (Ref)
Frequency of G	77 (25.7%)	65 (21.7%)	1.58 (0.39–2.84)	0.72
***GSTO1*(rs4925:C>A)**				
CC	87 (58%)	89 (59.4%)	1 (Ref)	
CA	53 (35%)	47 (31.3%)	1.39 (0.29–1.97)	0.47
AA	10 (7%)	14 (9.3%)	0.55 (0.02–2.72)	0.69
Frequency of C	227 (75.7%)	225 (75%)	1 (Ref)
Frequency of A	73 (24.3%)	75 (25%)	0.77 (0.09–2.18)	0.84
***GSTO2 (rs156697:A>G)***				
AA	59 (39%)	85 (56.7%)	1 (Ref)	
AG	69 (46%)	53 (35.3%)	2.58 (1.31–4.86)	**0.01** ^**^
GG	22 (15%)	12 (8.0%)	3.13 (1.55–6.04)	**0.01** ^**^
Frequency of A	187 (62%)	223 (74.3%)	1 (Ref)
Frequency of G	113 (38%)	77 (25.7%)	2.37 (1.09–4.33)	0.001^**^

**Statistically significant values.

The odds ratio is calculated after adjusting age, gender, and dwelling.

For *GSTO2* (rs156697: A>G) SNP, the frequencies of AA, AG, and GG genotypes in ALL patients were 39%, 46%, and 15%, respectively, while the respective frequencies of the same genotypes were 56.7%, 35.3%, and 8.0% in controls. A significant difference in the distribution of heterozygous *GSTO2-*AG (*p* = 0.01) and homozygous variant genotype *GSTO2-*GG (*p* = 0.01) was observed between ALL cases and controls. Furthermore, the frequency of rs156697 G allele was higher in patients as compared to controls (38% vs 25.7%) and along with *GSTO2*-AG and GG sequence variants showed a significant association with B-ALL risk (OR = 2.37, 95% CI = 1.09–4.33, *p* = 0.001) (AG; OR = 2.48, 95% CI = 1.31–4.86, *p* = 0.01) (OR = 3.13, 95% CI = 1.55-6.04, *p* = 0.01) (**Table 2**).

### Combined GST Genotypes/Haplotypes and B-ALL Risk

The SNPs were stratified together to observe the cumulative combinational effect of genotypes on ALL risk. When the frequencies of *GSTT1/GSTM1* genotypes were combined and their impact on ALL susceptibility was observed, *GSTT1*_null_ in combination with *GSTM1*_null_ (OR = 4.11, 95% CI = 1.16–7.62, *p* = 0.011) significantly associated with the increased risk of ALL. On combined genotypic analysis of *GSTT1* and *GSTP1* polymorphisms, a significantly higher frequency of *GSTT1*_null_/*GSTP1*-AG (16%) was observed in cases compared to 3.3% in controls and associated with increased ALL risk (OR = 4.93, 95% CI = 1.76–9.61, *p* = 0.003). Furthermore, *GSTM1* and *GSTP1* combined genotypes showed no association with the risk of developing ALL (**Table 3**).

**Table 3:** Combined genotype and haplotype analysis of *GST* polymorphisms in ALL patients and controls.

Combined Genotypes		ALL Cases (*n* = 150) (%)	Controls (*n* = 150) (%)		OR (95% CI)		*p*-value
*GSTT1*	*GSTM1*						
Present	Present	63 (42)	74 (49.3)		1 (Ref)		–
Present	Null	27 (18)	53 (35.3)		0.29 (0.06–2.35)		0.13
Null	Present	44 (29)	19 (12.7)		2.18 (1.16–3.51)		0.39
Null	Null	16 (11)	4 (2.7)		4.11 (1.16–7.26)		0.011^**^
***GSTT1***	***GSTP1***						
T1(+)	AA	60 (40)	74 (49.4)		1 (Ref)		–
T1(+)	AG	23 (15)	47 (31.3)		0.93 (0.47–1.92)		0.21
T1(+)	GG	7 (5)	5 (3.9)		1.21 (0.17–3.99)		0.37
T1(-)	AA	28 (19)	17 (11.4)		1.73 (0.88–6.39)		0.09
T1(-)	AG	24 (16)	5 (3.3)		4.93 (1.76–9.61)		**0.0003** ^**^
T1(-)	GG	8 (5)	2 (1.3)		4.56 (1.62–7.63)		0.07
***GSTM1***	***GSTP1***						
M1(+)	AA	72 (48)	56 (37.3)		1 (Ref)		–
M1(+)	AG	33 (22)	34 (22.7)		0.97 (0.63–1.92)		0.29
M1(+)	GG	2 (1)	4 (2.7)		0.99 (0.25–2.73)		0.62
M1(-)	AA	16 (11)	36 (24)		0.61 (0.03–1.36)		0.13
M1(-)	AG	14 (9)	17 (11.3)		0.81 (0.11–1.85)		0.68
M1(-)	GG	13 (9)	3 (2.0)		2.44 (1.14–8.55)		0.47
**Haplotypes**							
***GSTO1***	***GS2O2***	**Frequency**		**OR (95% CI)**		***p*-value**	
C	A	0.510		1 (Ref)		–	
C	G	0.243		1.52 (0.13–2.81)		**0.004** ^**^	
A	A	0.173		0.89 (0.56–1.40)		0.61	
A	G	0.073		0.81 (0.38–1.72)		0.59	

**Statistically significant values.

The odds ratio is calculated after adjusting age, gender, and dwelling. T1(+): GSTT1_Present;_ T1(-): GSTT1_Null_; M1(+): GSTM1_Present;_ M1(-): GSTM1_Null_.

Since *GSTO1* (rs4925: C>A) and *GSTO2* (rs156697: A>G) SNPs are linked, we performed haplotype analysis to estimate cumulative effect of two sequence variations on the risk of ALL. Among the haplotypes, only CG haplotype associated significantly with risk of B-ALL (OR = 1.52, 95% CI = 0.13–2.81, *p* = 0.004) (**Table 3**).

### GST Genotypes and Outcome for B-ALL patients

**Figure 2:**
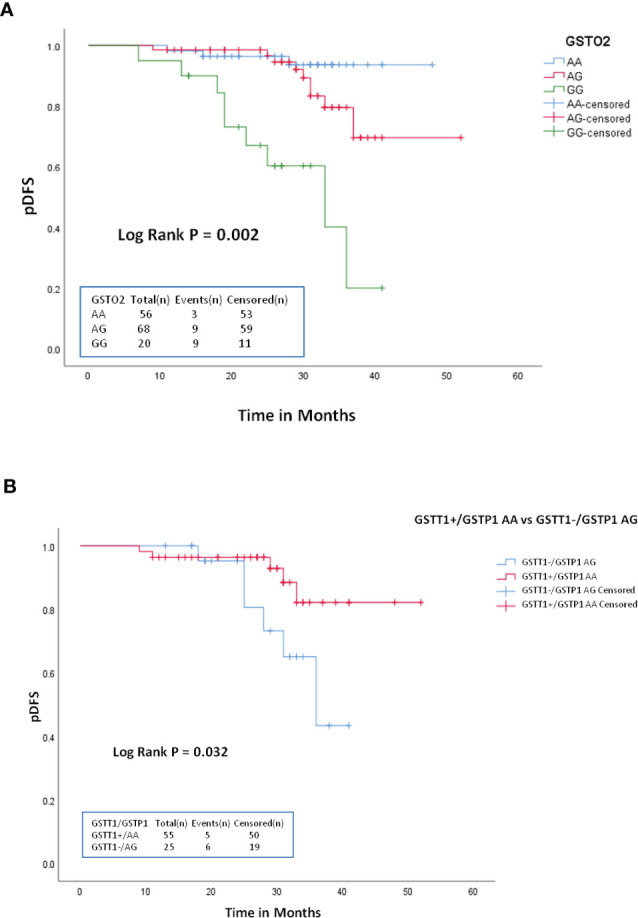
Kaplan-Meier survival plots stratified according to: **(A)** GSTO2-AA vs GSTO2-AG and GSTO2-GG **(B)** GSTT1+/GSTP1-AA vs GSTT1+/GSTP1-AG with respect to probability of disease free survival (DFS) during treatment.

Association of GST genotypes with therapeutic outcome of the patient group was evaluated by Kaplan–Meier survival plots. The median follow-up time was 19.2 months (range 7–52 months). Of the 150 patients, 123 (82%) achieved remission and 21 (14%) patients relapsed. Also, six patients died at different treatment stages during the study. None of the GST SNPs showed any association with the overall survival. Also, no association with DFS was found for *GSTT1*, *GSTM1*, *GSTP1*, or *GSTO1* genotypes However, Kaplan–Meier survival plots revealed significantly inferior 3-year probability of DFS (p3yDFS) of 23% for *GSTO2*-GG variant genotype carriers compared to 78.2% and 93.7% for patients who carried *GSTO2*-AA or AG genotypes (log-rank *p* = 0.002) (**Figure 2A**). Multivariate analysis confirmed *GSTO2*-GG variant genotype as an independent poor prognostic factor for DFS (HR = 4.51, 95% CI: 0.81-11.8, *p* = 0.034) as it conferred greater than fourfold increased relapse risk in B-ALL cases after adjusting other test variables like age, gender, baseline TLC, and ALL risk groups (**Table 4**).

**Table 4:** Multivariate analysis for disease-free survival (DFS) of ALL patients according to different GST genotypes.

Variable	Hazard Ratio	95.0% CI	*p*-value
***GSTT1***	0.68	0.19–2.40	0.5
***GSTM1***	0.33	0.06–1.57	0.1
***GSTP1*-AG**	0.58	0.14–2.26	0.4
***GSTP1*-GG**	0.30	0.07–13.1	0.4
***GSTO1*-CA**	0.87	0.22–3.44	0.8
***GSTO1*-AA**	1.24	0.15–10.1	0.8
***GSTO2*-AG**	0.30	0.08–1.10	0.07
***GSTO2*-GG**	4.51	0.81–11.8	**0.034** ^**^

**Statistically significant values.

All predictor variables like age, gender, dwelling, baseline WBC counts, risk groups, and various GST polymorphisms were assessed together by multivariate analysis to observe whether their individual effect as ascertained by univariate analysis would be impacted in combination or they would retain their effect and emerge as an independent risk factor.

Furthermore, among various G*ST* combined genotypes, only *GSTT1*_null_/*GSTP1*-AG showed significant influence on DFS as patients carrying this combined genotype had significantly reduced p3yDFS of 42.7% compared to 81.3% for *GSTT1*_present_/*GSTP1*-AA carriers (log-rank *p* = 0.032) (**Figure 2B**).

## Discussion

Initiation of leukemogenesis is likely caused by multiple factors; nevertheless, the exact mechanisms underlying remains poorly understood. Hereditary differences in the expression and activity of human GSTs have been reported, and altered GST enzymatic activity is associated with different types of cancer (27, 28). This is the first of its kind case–control study from North India to evaluate the influence of GST polymorphisms particularly *GSTO1* (rs4925: C>A) and *GSTO2* (rs156697: A>G) on the susceptibility and outcome of B-ALL.

Studies have shown that reduced glutathione with electrophilic compounds is highly soluble in water, permitting their elimination, and this detoxification activity prevents cells from DNA damage, genomic instability, and cancer development (9, 10). GSTs have the ability to modulate the non-enzymatic proteins and signalling pathways that control cell proliferation, differentiation, and apoptosis (29). Various types of *GSTs* translate internal and external carcinogenic compounds and ROS to non-toxic substances. *GSTM1* and *GSTT1* null are considered loss-of-function mutations as they involve the loss of structural homozygosity and predominantly lead to loss in the corresponding enzyme activity (6). In the *GSTP1* gene, the common A to G transition at 1578 nucleotide position within exon 5 reverts the isoleucine residue (A allele) with valine (G allele) at codon 105 and affects the conjugative ability of reducing glutathione (30). The presence of the G allele decreases the enzymatic efficiency of GST and in turn decreases the antioxidant capacity and increases the oxidative stress and subsequent cellular damage (30). This polymorphism results in reduction of the enzyme activity and is associated with the presence of a high level of hydrophobic DNA adducts (31, 32). The current study did not find any significant association of *GSTM1* and *GSTP1* polymorphisms with the risk of ALL, whereas the frequency of *GSTT1*_null_ genotype was significantly higher in ALL cases and associated with nearly threefold risk of ALL (*p* = 0.0001). Consistent with our results, a meta-analysis of 30 case–control studies suggested *GSTT1*_null_ genotype as a risk factor for ALL (7). A recent study showed further consistency with this scenario where *GSTT1*_null_ genotype significantly increased the ALL risk but *GSTM1*_null_ did not (6). The results of our study are further strengthened by studies from the subcontinent and across the globe (33–35). Furthermore, our study is in sync with many other studies that also showed no significant influence of *GSTP1* (rs1695: A>G) polymorphic variants on ALL risk (36–38).

GST omega is a structurally and functionally distinct class among the GST superfamily and its sequence variants have been recognized to decrease functional capability of the enzyme against cellular oxidative stress by about 75% (39–41). *GSTO* participates in cellular signaling and overexpression of *GSTO* has been reported to be linked to the induction of apoptosis involving the development of cancer (42). Recent studies have demonstrated two SNPs in *GSTO* gene [*GSTO1* (rs4925: C>A) and *GSTO2* (rs156697: A>G)] being associated with different cancer types, and majority of these studies have substantiated *GSTO2* as a risk factor for various solid tumors (15, 43, 44). Only two studies to date have evaluated the association of *GSTO1/2* SNPs with childhood ALL (15, 22). One of the studies observed *GSTO1* association with ALL susceptibility and *GSTO2* associated significantly with the high-risk group while another study did not find any such significance (15, 22). The current study, therefore, is the first from the subcontinent to report the impact of *GSTO1/2* SNPs on susceptibility and therapeutic outcome of B-ALL. We observed no association of *GSTO1* (rs4925:C>A) with B-ALL, whereas the heterozygous (AG) and homozygous variant (GG) genotypes of *GSTO2* depicted a significant association (*p* < 0.05). A recent meta-analysis including 4770 cases and 5701 controls exploring the association between *GSTO* polymorphisms and cancer risk showed no significant association between the *GSTO1* polymorphism and cancer susceptibility whereas the “GG” genotype of the *GSTO2* polymorphism was observed to increase the risk of overall cancer and breast cancer (45).

The study of a disease with respect to different genetic variants in the same pathway pertaining to similar loci strengthens their analytical influence rather than considering single gene variants. Based on this hypothesis and evident GST gene interactions, we analyzed the combined effect of *GSTT1, GSTM1*, and *GSTP1* genotypes on ALL (46). Among *GSTT1* and *GSTM1* combinations, *GSTT1*/*GSTM1* double null associated with more than fourfold risk for ALL respectively (*p* = 0.011). In agreement with our findings, Moulik et al. reported a significant >3-fold increased risk of ALL associated with *GSTT1*/*GSTM1* double null genotype whereas another study on ALL reported no significant association (6, 47). Furthermore, in tune with our study, most studies have consensus of a strong association between *GSTM1/GSTT1* double null genotype and risk of ALL in Asian population (6, 48). Similarly, on analyzing the relation of *GSTP1* with *GSTT1* and *GSTM1* genotypes, the presence of *GSTT1*_null_ */GSTP1*-AG genotype associated with nearly fivefold increased risk of ALL (*p* = 0.0003).

As *GSTO1* (rs4925:C>A) and *GSTO2* (rs156697:A>G) are linked, we aimed to analyze the influence of *GSTO1/2* haplotypes on B-ALL risk. In the present study, only CG haplotype revealed significant association with risk of B-ALL (*p* = 0.004). At present, only two studies have reported the relationship between rs4925: C>A and rs156697: A>G SNPs of *GSTO* gene and ALL but none of them studied the association of *GSTO1/2* haplotypes on ALL risk (15, 19). As no study could be traced to have investigated the *GSTO1/2* haplotypes in ALL, the results of this parameter could not be compared for conclusive remarks.

SNPs in genes that encode metabolizing enzymes and drug transporters may alter drug efficacy and, therefore, can influence treatment response (6, 47, 49). The reports about the outcome of the patients with respect to GST genotypes across the globe have been controversial. The analysis of different factors that influence the clinical outcome of ALL patients in our study showed that only *GSTO2* SNP seemed to impact the survival outcome of patients as carriers of its homozygous variant GG had significantly lower DFS (log-rank *p* = 0.002). Multivariate hazard analysis also proved *GSTO2*-GG as an independent poor prognostic factor of DFS (*p* = 0.034). This finding makes the *GSTO2* gene a plausible factor to predict the response outcome of ALL patients but needs authentication on large sample size. The two studies reported to date regarding the association of *GSTO* polymorphism with the susceptibility of childhood ALL have not analyzed their impact on therapeutic outcome (15, 22), and in this regard, our study is the first to report the impact of *GSTO1* (rs4925: C>A) and *GSTO2* (rs156697: A>G) SNPs on the therapeutic outcome of ALL patients. Furthermore, no significant correlation between the allelic variants of GST SNPs and treatment arm with respect to survival outcomes in ALL patients was observed. Several studies in accordance with our report also accounted for no influence of *GSTT1*, *GSTM1*, and *GSTP1* genotypes on the outcome of ALL patients (33, 44, 50), whereas the study by Takanashi et al. found significantly higher risk of relapse in Japanese BCP-ALL patients that carried *GSTM1*_null_ and *GSTT1*_null_ genotypes (51). In the present study, among various G*ST* combined genotypes, only *GSTT1*_null_/*GSTP1*-AG significantly influenced the DFS (log-rank *p* = 0.037) but did not affect the OS of ALL patients. Similar to our observations, the study by Suneetha et al. substantiated *GSTP1* as an independent poor prognostic factor where both homozygous and heterozygous variants of *GSTP1* associated with poor survival outcome in ALL patients (47).

## Conclusion

We conclude that *GSTT1*_null_ and *GSTO2* (AG and GG) genotypes seem to increase the risk of B-ALL, whereas *GSTM1*, *GSTP1*, and *GSTO1* polymorphisms had no role in the pathogenesis or therapeutic outcome of B-ALL. Also, results suggest a correlation of *GSTO2-GG* polymorphic variant with treatment outcome. The limitations of the present study remain due to the slightly lower sample size. Nonetheless, more studies considering the genetic variability in GSTs with increased sample sizes in different ethnicities are needed to decipher new susceptibility and prognostic markers in ALL to optimize personalized therapeutic approach.

## Data Availability Statement

The original contributions presented in the study are included in the article/**Supplementary Material**. Further inquiries can be directed to the corresponding author.

## Ethics Statement

The studies involving human participants were reviewed and approved by the Institutional Ethics Committee of Sher-i-Kashmir Institute of Medical Sciences. Written informed consent to participate in this study was provided by the participants’ legal guardian/next of kin.

## Author Contributions

SB conceptualized and designed the study, wrote the manuscript, and did lab work. AP performed the statistical analysis and wrote sections of the manuscript. SAG and JB provided patient samples. AG did lab work. ZS supervised the study, provided logistics, and proofread and submitted the manuscript. SG, HE-S, AK, and SM revised, corrected, and proofread the manuscript. All authors contributed to the article and approved the submitted version.

## Conflict of Interest

The authors declare that the research was conducted in the absence of any commercial or financial relationships that could be construed as a potential conflict of interest.

## Publisher’s Note

All claims expressed in this article are solely those of the authors and do not necessarily represent those of their affiliated organizations, or those of the publisher, the editors and the reviewers. Any product that may be evaluated in this article, or claim that may be made by its manufacturer, is not guaranteed or endorsed by the publisher.
