# Supplementary material for: GSTT1 null and rs156697 Polymorphism in GSTO2 Influence the Risk and Therapeutic Outcome of B-Acute Lymphoblastic Leukemia Patients
Source: Front Oncol. 2021 Oct 14;11:714421. doi: 10.3389/fonc.2021.714421 (PMC8552530; doi:10.3389/fonc.2021.714421)
Supplement: Supplementary file 5 [file Table_1.docx]

| **Gene** | **Primer Sequence** | **Thermal conditions** | **Amplicon Size (bp)** |
| --- | --- | --- | --- |
| ***GSTM1*** | F:5´-GTTGGGCTCAAATATACGGTGG-3´  R:5´-GAACTCCCTGAAAAGCTAAAGC-3´ | 94°C for 5 min, 94°C for 35s, 54°C for 35 s and 72°C for 35 s (35 cycles), 72°C for 7 min. | 240bp |
| ***GSTT1*** | F:5´-TTCCTTACGGTCCTCACATCTC-3´  R:5´-TCACCGGATCATGGCCAGCA-3´ | 94°C for 5 min, 94°C for 35s, 54°C for 35 s and 72°C for 35 s (35 cycles), 72°C for 7 min. | 450bp |
| ***GSTP1***  (Ile105Val) | F:5´-ACCCCAGGGCTCTATGGGAA-3´  R:5´TGAGGGCACAAGAAGCCCCT-3´ | 95°C for 4 min, 94°C for 25s, 55°C for 25 s and 72°C for 30 s (35 cycles), 72°C for 5 min. | 176bp |
| ***GSTO1***  ***(Ala140Asp)*** | F:5´-GAACTTGATGCACCCTTGGT-3´  R:5´-TGATAGCTAGGAGAAATAATTAC-3´ | 95°C for 4 min, 94°C for 25s, 60°C for 25 s and 72°C for 30 s (35 cycles), 72°C for 5 min. | 254bp |
| ***GSTO2***  ***(Asp142Aspr)*** | F:5´-AGGCAGAACAGGAACTGGAA-3´  F:5´-GAGGGACCCCTTTTTGTACC-3´ | 95°C for 4 min, 94°C for 25 s,60°C for 25 s and 72°C for 30 s (35 cycles), 72°C for 5 min. | 185bp |
| ***β-Actin*** | F:5´-TGACGGGGTCACCCACACTGT-3´  R:5´-CTAGAAGCATTTGCGGTGGAC-3´ | 94°C for 5 min, 94°C for 35s, 54°C for 35 s and 72°C for 35 s (35 cycles), 72°C for 7 min. | 868bp |

**Supplementary Table: Sequence of primers and thermal conditions for amplification of various GST SNPs along with their respective amplicon size**
